# SARS-CoV-2 drives NLRP3 inflammasome activation in human microglia through spike protein

**DOI:** 10.1038/s41380-022-01831-0

**Published:** 2022-11-01

**Authors:** Eduardo A. Albornoz, Alberto A. Amarilla, Naphak Modhiran, Sandra Parker, Xaria X. Li, Danushka K. Wijesundara, Julio Aguado, Adriana Pliego Zamora, Christopher L. D. McMillan, Benjamin Liang, Nias Y. G. Peng, Julian D. J. Sng, Fatema Tuj Saima, Jenny N. Fung, John D. Lee, Devina Paramitha, Rhys Parry, Michael S. Avumegah, Ariel Isaacs, Martin W. Lo, Zaray Miranda-Chacon, Daniella Bradshaw, Constanza Salinas-Rebolledo, Niwanthi W. Rajapakse, Ernst J. Wolvetang, Trent P. Munro, Alejandro Rojas-Fernandez, Paul R. Young, Katryn J. Stacey, Alexander A. Khromykh, Keith J. Chappell, Daniel Watterson, Trent M. Woodruff

**Affiliations:** 1 School of Biomedical Sciences, Faculty of Medicine, University of Queensland, St Lucia, QLD 4072 Australia; 2 School of Chemistry and Molecular Biosciences, University of Queensland, St Lucia, QLD 4072 Australia; 3 Australian Institute for Bioengineering and Nanotechnology, The University of Queensland, St Lucia, QLD 4072 Australia; 4 Institute of Medicine, Faculty of Medicine, Universidad Austral de Chile, Valdivia, Chile; 5 Molecular Medicine Laboratory, Medical School, Universidad de Costa Rica, San Pedro, Costa Rica; 6 Australian Infectious Disease Research Centre, Global Virus Network Centre of Excellence Brisbane, Brisbane, QLD 4072 and 4029 Australia; 7 Queensland Brain Institute, University of Queensland, St Lucia, QLD 4072 Australia; 8 Present Address: Vaxxas Pty. Ltd., Woolloongabba, QLD 4102 Australia

**Keywords:** Neuroscience, Cell biology

## Abstract

Coronavirus disease-2019 (COVID-19) is primarily a respiratory disease, however, an increasing number of reports indicate that SARS-CoV-2 infection can also cause severe neurological manifestations, including precipitating cases of probable Parkinson’s disease. As microglial NLRP3 inflammasome activation is a major driver of neurodegeneration, here we interrogated whether SARS-CoV-2 can promote microglial NLRP3 inflammasome activation. Using SARS-CoV-2 infection of transgenic mice expressing human angiotensin-converting enzyme 2 (hACE2) as a COVID-19 pre-clinical model, we established the presence of virus in the brain together with microglial activation and NLRP3 inflammasome upregulation in comparison to uninfected mice. Next, utilising a model of human monocyte-derived microglia, we identified that SARS-CoV-2 isolates can bind and enter human microglia in the absence of viral replication. This interaction of virus and microglia directly induced robust inflammasome activation, even in the absence of another priming signal. Mechanistically, we demonstrated that purified SARS-CoV-2 spike glycoprotein activated the NLRP3 inflammasome in LPS-primed microglia, in a ACE2-dependent manner. Spike protein also could prime the inflammasome in microglia through NF-κB signalling, allowing for activation through either ATP, nigericin or α-synuclein. Notably, SARS-CoV-2 and spike protein-mediated microglial inflammasome activation was significantly enhanced in the presence of α-synuclein fibrils and was entirely ablated by NLRP3-inhibition. Finally, we demonstrate SARS-CoV-2 infected hACE2 mice treated orally post-infection with the NLRP3 inhibitory drug MCC950, have significantly reduced microglial inflammasome activation, and increased survival in comparison with untreated SARS-CoV-2 infected mice. These results support a possible mechanism of microglial innate immune activation by SARS-CoV-2, which could explain the increased vulnerability to developing neurological symptoms akin to Parkinson’s disease in COVID-19 infected individuals, and a potential therapeutic avenue for intervention.

## Introduction

Neuroinflammation is a hallmark of neurodegenerative diseases. A variety of stimuli within the central nervous system (CNS), including pathogens, injury, toxic metabolites, and protein aggregates among others, can lead to the activation of the innate immune response mainly through microglial activation. When chronically activated, this defence mechanism creates a proinflammatory environment that drives neurodegeneration [1, 2]. Microglia are resident populations of macrophages in the CNS that respond to pathogen-associated molecular patterns (PAMPs) and host- or environment-derived danger-associated molecular patterns (DAMPs) to drive innate immune responses and inflammation within the brain. Recent evidence has highlighted the role of intracellular protein complexes, known as the inflammasomes, in CNS innate immunity.

These complexes mediate the response to PAMPs and DAMPs, leading to the generation of IL-1β, IL-18 and ultimately cellular pyroptosis, which can aid in the elimination of invading pathogens, clearance of damaged cells, and promotion of tissue repair [3]. The NLR family pyrin domain containing 3 (NLRP3) inflammasome is a key inflammasome expressed by microglia [2], and is activated by multiple protein aggregates associated with neurodegenerative disease including α-synuclein in Parkinson’s disease (PD), amyloid-β in Alzheimer’s disease, and TDP43 and SOD1 aggregates in amyotrophic lateral sclerosis [4–6]. Microglial NLRP3 inflammasome can also be activated by a variety of pathogenic viruses with neurotropism such as Zika virus and Japanese Encephalitis virus (JEV) [7, 8]. The NLRP3 inflammasome is comprised of the NLRP3 protein, the adaptor molecule apoptosis-associated speck-like protein containing a CARD (ASC), and caspase-1. Activation of NLRP3 is a two-step process; a priming step usually mediated through a Toll-like receptor involves NF-kB-dependent induction of both NLRP3 and pro-IL-1β, whereas the triggering step leads to oligomerisation of NLRP3, recruitment of ASC, and recruitment and activation of caspase-1. Active caspase-1 then cleaves pro-IL-1β and pro-IL-18 into their active forms, and initiates pyroptotic cell death [3].

The hypothesis that viral infections can accelerate neurodegeneration is gaining attention with relevance to the current COVID-19 pandemic [9]. It has become clear that severe acute respiratory syndrome coronavirus 2 (SARS-CoV-2) can affect multiple organs and tissues including the brain [10, 11]. Post-mortem analysis of brains obtained from deceased SARS-CoV-2 patients showed extensive microglial activation with pronounced neuroinflammation in the brainstem [12, 13]. This extensive microglial activation upon SARS-CoV-2 infection has been also reported using different in vivo models including K18 hACE2 mice [14], Golden Syrian hamsters [15], and non-human primates [16].

Moreover, accumulating evidence shows that acute and sub-acute neurological complications of SARS-CoV-2 infections are reported in up to 85% of patients not only with severe COVID-19, but also in mildly symptomatic or asymptomatic patients [17, 18]. These manifestations include headache, dizziness, impaired consciousness, encephalopathy, delirium, confusion, seizure, gait difficulties, cerebrovascular events, and post-infectious autoimmunity [19]. Furthermore, psychiatric, and neuropsychiatric presentations have been linked to SARS-CoV-2 infection, with COVID-19 patients presenting with depression, anxiety, mood, and psychotic disorders [20, 21].

Peripheral disorders include Guillain-Barre-syndrome, myositis-like muscle injury, and notably, up to 65% of COVID-19 affected patients reported decreased sense of smell or hyposmia [18], which also is a common pre-motor symptom in PD [22]. Additionally, reported cases of PD linked to COVID-19 [23–25], have triggered attention to evaluating SARS-CoV-2 infections and their impact on PD [26, 27]. However, the specific mechanism of how SARS-CoV-2 could increase the risk of developing neurological manifestations, and potentially PD, and how this infection could possibly impact synucleinopathy has not been demonstrated.

Here, we used the K18 hACE2 transgenic mouse COVID-19 preclinical model [28] to assess microglial NLRP3 inflammasome expression in the brain upon SARS-CoV-2 infection, and a human monocyte-derived microglia (MDMi) cellular model to assess inflammasome activation in response to SARS-CoV-2 and its spike protein, and the consequences of this exposure in the presence of α-synuclein protein aggregate fibrils. We determined that SARS-CoV-2 presence in the brains of hACE2 mice is associated with marked microglial inflammasome activation, which was blocked by a specific NLRP3 inhibitor. Mechanistically, we show that SARS-CoV-2 isolates, as well as spike protein alone, can both prime and activate the NLRP3 inflammasome in human microglia through NF-κB and ACE2. Microglia exposed to SARS-CoV-2, or its spike protein, also potentiated α-synuclein mediated NLRP3 activation, indicating a possible mechanism for COVID-19 and increased vulnerability to developing movement disorders in certain infected individuals.

## Materials and methods

### Study design

Studies were primarily designed: (i) to determine whether increased microglial NLRP3 inflammasome activation occurs in vivo after SARS-CoV-2 infection; (ii) to determine whether increased NLRP3 inflammasome activation occurs in MDMi exposed to SARS-CoV-2 isolates; and (iii) to evaluate this activation in the presence of preformed fibrils of α-synuclein (Syn).

### Ethics and biological safety

Ethical approval for collecting and utilising human donor blood was obtained from The University of Queensland Human Research Ethics Committee (HREC approval #2020000559). All experiments with pathogenic SARS-CoV-2 were conducted under a certified biosafety level-3 (BSL-3) conditions in the School of Chemistry and Molecular Biosciences at The University of Queensland (SCMB-UQ). All personnel used powered air purifying respirator (PAPR; SR500 Fan Unit) as a respiratory protection at all the time within the facility. Surface disinfection was performed using 70% ethanol, while liquid and solid waste were steam-sterilized by autoclave. This work was approved by the Institutional Biosafety Committee from The University of Queensland (UQ) (UQ IBC, approvals IBC/390B/SCMB2020, IBC/1301/SCMB/2020, IBC/376B/SBMS/2020 and IBC/447B/SCMB/2021).

### Animals and virus infection

The use of animals was approved by the University of Queensland Animal Ethics Committee under the project number: 2021/AE001119. Mice were housed using IsoCage N-Biocontainment System (Tecniplast, USA) where each cage is supplied with an HEPA filter to prevent viral transmission between cages. Each cage receives individual ventilation that allows an environment maintained under 65-70% humidity and an ambient temperature of 20–23 °C. Mice were kept within an animal BSL-3 facility under a 12-h light/dark cycle with food and water provided ad libitum. Six-week-old female mice carrying the human ACE2 gene under the control of the keratin 18 promoter (K18-hACE2) on C57BL/6J background were purchased from Animal Resource Centre (ARC) in Australia and randomly assigned to cages. Group sizes were determined based on our previous studies [4, 29], and contained *n* = 6 for clinical scoring and *n* = 4 for tissue collection. Mice were anesthetized with ketamine (100 mg/kg) and xylazine (10 mg/kg), followed by intranasal inoculation of 20 µL of the ancestral SARS-CoV-2 (Wu strain) at 5 x 10^3^ FFU per mouse, or RPMI additive free medium for the uninfected control group. Mice were monitored daily for clinical symptoms and weight loss for up to 12 days post infection. Clinical scoring included: no disease (0); hindlimb weakness, away from littermates, ruffled fur (0.5–1); partial hindlimb paralysis, limping, hunched, reluctant to move (1.5–2); and complete paralysis of hindlimb, severely restricted mobility, severe distress, or death (2.5–3). For treatment studies, mice were dosed daily with MCC950 (Cat# inh-mcc, Invivogen) via oral gavage (20 mg/kg) [4], or vehicle (water), 24 h after infection for 11 days. Study investigators (EA and AAA) were blinded to the treatment groups by using colour-coded drug vials that were generated by an independent investigator (JDL).

### Murine immunofluorescence studies

Mice were deeply anesthetized with a mixture of xylazine (10 mg/kg), and Ketamine (100 mg/kg) and then perfused transcardially with PBS. Brains were removed, post-fixed in 4% paraformaldehyde (PFA) overnight, followed by sucrose gradient, and 30-micron sections were cut using a cryostat (Thermo Cryostar NX70). Sections were processed for immunofluorescence as previously described [4]. Mild antigen retrieval was performed by incubating sections in citrate buffer (10 mM sodium citrate, pH 7.5) for 30 min at 60 °C. Sections were then washed several times in PBS and blocked with PBS containing 2% BSA, 2% normal donkey serum, 0.1% Triton X-100 and 0.05% tween-20 for 2 h at room temperature. Sections were incubated with the respective primary antibodies: Nucleocapsid C2 [29]; ASC (Cat #AG-25B-0006-C100, Adipogen); NLRP3 Cryo-2 (Cat# AG-20B-0014-C100,Adipogen); Iba1 (Cat# 019-19741,Wako); or TMEM119 (Cat# 2542,Synaptic systems) overnight at 4 °C in PBS containing 2% BSA and 2% donkey serum. After, 5 washes in PBS, sections were incubated with Alexa dye-conjugated secondary antibodies for 2 h at room temperature. The nucleus was labelled with DAPI stain and slides mounted with Prolong Gold antifade mounting medium (Invitrogen) according to manufacturer’s instructions. Samples were visualised using a slide scanner microscope (Zeiss AxioScan Z1 Fluorescent imager).

### Mouse brain quantitative real-time PCR

Total RNA was isolated from the whole brain of uninfected and SARS-CoV-2 (Wu strain) infected K18-hACE2 mice (*n* = 4–9) using RNeasy Lipid Tissue extraction kit according to manufacturer’s instructions (QIAGEN, CA, USA). The total RNA was purified from genomic DNA contamination using Turbo DNase treatment (Ambion, NY, USA), then converted to cDNA using AffinityScript cDNA synthesis kit according to manufacturer’s instructions (Agilent Technologies, CA, USA). Commercially available gene specific TaqMan probes for Aif1 (Mm00479862_g1), Pycard (Mm00445747_g1), and Casp1 (Mm00438023_m1) were used to amplify target gene of interest (Applied Biosystems, MA, USA). Relative target gene expression to geometric mean of reference genes Gapdh (Mm99999915_g1) and Actb (Mm02619580_g1) was determined using this formula: 2^-∆CT^ where ∆CT = (Ct_(Target gene)_ – Ct_(Gapdh and Actb)_). Final measures are presented as relative levels of gene expression in SARS-CoV-2 (Wu strain) infected K18-hACE2 mice compared with expression in uninfected K18-hACE2 mice. Probe set was tested over a serial cDNA concentration for amplification efficiency. Negative controls included no reverse transcription and water as a no template control. All samples were run in triplicate.

### Cells lines

Vero E6 cells (African green monkey kidney cell clones), Caco2 (human colorectal adenocarcinoma cells), and human embryonic kidney 293T cells (HEK293T) were maintained in Dulbecco’s Modified Eagle Medium (DMEM) supplemented with 10% heat inactivated foetal calf serum (FCS) (Bovogen, USA), penicillin (100 U/mL) and streptomycin (100 μg/mL) (P/S) and maintained at 37 °C with 5% CO_2_. All cell lines were verified to be mycoplasma free by first culturing the cells in antibiotic-free media and then subjected to a mycoplasma tested using MycoAlert™ PLUS Mycoplasma Detection Kit (Lonza, UK).

### Generation of human Monocyte-Derived Microglia (MDMi)

Monocytes were isolated from healthy donor blood collected into lithium heparin vacutainer tubes (Becton Dickinson) by a qualified phlebotomist following informed consent, or from buffy coats obtained from Australian Red Cross Lifeblood as previously described [30]. Briefly, donor blood or buffy coat was diluted 1:1 with phosphate buffered saline (PBS) and transferred into sterile SepMate 50 (STEMCELL Technologies, BC, Canada) as per manufacturer’s instructions. Peripheral blood monocytes (PBMCs) were collected. Monocytes were positively selected from whole PBMCs using anti CD14^+^ microbeads (Miltenyi Biotec) and plated at the following densities per well: 1 × 10^5^ cells (96-well plate) and 3 × 10^5^ cells (24-well plate). To induce the differentiation of MDMi, we incubated monocytes under serum-free conditions using RPMI-1640 Glutamax (Life Technologies) with 1% penicillin/ streptomycin (Lonza) and Fungizone (2.5 μg/ml; Life Technologies) and a mixture of the following human recombinant cytokines: M-CSF (10 ng/ml; Preprotech, 300-25), GM-CSF (10 ng/ml; Preprotech,300-03), NGF-β (10 ng/ml; Preprotech, 450-01), MCP-1(CCL2) (100 ng/ml; Preprotech, 300-04), and IL-34 (100 ng/ml; Preprotech, 200-34-250) under standard humidified culture conditions (37 °C, 5% CO_2_) for up to 14 days. Differentiation of PBMCs into MDMi was confirmed by western blot and immunofluorescence for microglial markers compared to monocyte-derived macrophages (MDM).

### MDMi treatments

For inflammasome activation experiments, MDMi were primed with 200 ng/ml of ultrapure LPS (*E.Coli* 0111:B4, Invivogen) for 3 h or 50 μg of S-clamp or F-clamp for 6 h. Cells were washed in after priming to remove residual LPS or S-Clamp and cells were stimulated with conventional NLRP3 inflammasome activators ATP (5 mM, Sigma) and nigericin (10 μM, Invivogen), or fibrillar α-synuclein (10 μM, Proteos), S-Clamp (2–50 μg) or SARS-CoV-2 isolates (MOI 0.1, 1) for the indicated time. For priming studies, MDMis were pre-treated with the NF-kB inhibitor, Bay 11-7082 (3 μM, Sigma), before stimulation with S-clamp and the addition of ATP, nigericin or α-synuclein. For inhibition studies, MCC950 (10 μM), VX-765 (20 μM, Invivogen) and MLN-4760 (1,10 μM, Sigma) were added after the priming step. At the end of treatment, the supernatant was collected and stored at −80 °C until analysis by enzyme-linked immunosorbent assay (ELISA) or western blotting.

### Quantification of caspase-1 mediated pyroptosis

At the end of each treatment, supernatants were collected and LDH release was quantified using an LDH assay kit (TOX7-Sigma) as per the manufacturer’s instructions. Caspase-1 dependent LDH release that was inhibited by the caspase-1 inhibitor VX-765 (20 μM), was used as a readout for pyroptosis as previously described [31].

### Viral isolate

SARS-CoV-2 were isolated from patient nasopharyngeal aspirates via inoculation in Vero E6 cells. An early Australian isolate hCoV-19/Australia/QLD02/2020 (QLD02) (GISAID Accession ID; EPI_ISL_407896) sampled on 30/01/2020 and named in this study as Wu. This virus isolated was provided by Queensland Health Forensic & Scientific Services, Queensland Department of Health as passage 2 in Vero E6 cells. Viral stocks (passage 3) were then generated on Vero E6 cells and stored at −80 °C. To ensure there was no passage-to-passage variation of viruses used in this study or loss of the spike furin cleavage on Vero E6 passaged SARS-CoV-2 isolate whole SARS-CoV-2 sequencing and variant bioinformatics analysis was conducted as per [32] to QLD02 isolate. Briefly, the nCoV-2019 Nanopore sequencing protocol v3 (Josh Quick, University of Birmingham) was used with minor modifications. RNA was isolated from cell culture supernatant, and cDNA generated using Protoscript II first-strand cDNA synthesis kit as per manufacturer’s protocol (New England Biolabs, USA). SARS-CoV-2 cDNA was subsequently amplified using ARTIC network v2 primers using two-step PCR amplification with Q5® High-Fidelity DNA Polymerase (New England Biolabs, USA). PCR fragments were purified using AMPure XP beads (Beckman Coulter, USA) and subjected to End Repair/dA-Tailing using the NEBNext® Ultra™ II Module (New England Biolabs, USA). Passage 3 of QLD02 sample was multiplexed using the Native Barcoding Expansion kit (EXP-NBD104, Oxford Nanopore, UK) and Ligation Sequencing Kit (SQK-LSK109, Oxford Nanopore, UK). Prepared libraries were then quantified and loaded into equimolar concentrations totalling 20 fmol into a Flongle flow cell (FLOFLGOP1, Oxford Nanopore, UK). Variant analysis was conducted using iVar (v1.2.2) [33] and depth of sequencing coverage and consensus positions were visualised and calculated using Integrative Genomics Viewer (Version: 2.7.0). Virus stock titre was determined by an immuno-plaque assay (iPA) as previously described [34].

### Growth kinetics

SARS-CoV-2 (Wu) replication kinetics was assessed on Vero E6, Caco2, mouse primary microglia and MDMi cells. Briefly, 5 x 10^5^ cells were seeded in 24-well plates 1 day before infection. Cells were infected at a MOI of 1 or 0.1 for 30 min at 37 °C. The monolayer was washed five times with 1mL of additive-free DMEM and finally incubated with 1 mL of DMEM (supplemented with 2% FCS and P/S) at 37 °C with 5% CO_2_. Infectious viral titres were assessed in samples harvested from supernatant at the time points; 0-, 1-, 2- and 3-days post-infection (dpi). The viral titre was determined by an immuno-plaque assay (iPA) on Vero E6 cells. Two independent experiments were performed with 2 technical replicates.

### Binding assay

Vero E6 and MDMi cells were inoculated with an MOI = 1 of SARS-CoV-2 (Wu) for 2 h at 4 °C and then, cells were washed 8 times with fresh cold media to remove unbound viruses. Cells were then harvested in TRI Reagent (Millipore, Sigma-Aldrich, Germany) and total RNA was purified and quantified by real time RT-PCR.

### RNA extraction

RNA was extracted using TRIzol (Thermo Fisher) plus an RNA extraction RNeasy Micro kit (Qiagen). Briefly, the aqueous phase containing the RNA was separated by adding chloroform to the TRIzol samples and centrifuging them for 30 min, 4 °C at 12,000 g. An equal volume of 70% ethanol was added to the isolated aqueous phase, mixed and then added to the RNeasy MinElute spin column. The following washes, DNase I treatment and elute steps were performed as described in the Qiagen RNeasy Micro Handbook. RNA samples were eluted in RNase free water.

### MDMi quantitative real-time PCR

One-step quantitative real-time PCR was performed in a QuantStudio 6 (Thermo Fisher) using GoTaq® Probe 1-Step RT-qPCR System (Promega). The CDC SARS-CoV-2 nucleoprotein N2 primer set was used for amplification. Forward: 5’-TTA CAA ACA TTG GCC GCA AA-3’, Reverse: 5’-GCG CGA CAT TCC GAA GAA-3’ and Probe: 5’-FAM-ACA ATT TGC CCC CAG CGC TTC AG-BHQ1-3’ [35]. The standard curve was done using the 2019-nCoV_N Positive Control nucleoprotein DNA (IDT). A fixed volume representing 1/16 of the total RNA contained in each sample (well) was added to the master mix. Total number of copies were calculated using a semi-log line regression using GraphPad Prism 8.0.1. The human ACE2 transcript variant 2 was amplified using the OriGene primer set Forward: 5’-TCC ATT GGT CTT CTG TCA CCC G-3’ and Reverse: 5’-AGA CCA TCC ACC TCC ACT TCT C-3’. HPRT was used as the control housekeeping gene using primers, Forward: 5’-TCA GGC AGT ATA ATC CAA AGA TGG T-3’ and Reverse: 5’-AGT CTG GCT TAT ATC CAA CAC TTC G-3’. The relative expression is equal to the 2^(-∆*Ct*)^.

### Pseudo-virus entry assay

Pseudo-virus particles for SARS-CoV-2 were generated by using a lentiviral-based pseudo-particles system as previously described [36]. Briefly, HEK293T cells were co-transfected with the following plasmids: 1 μg of p8.91 (encoding a second-generation lentiviral packaging plasmid for HIV-1 gag-pol), 1.5 μg of pCSFLW (firefly luciferase reporter gene) and, 1 μg of plasmid encoding SARS-CoV2 spike (Wu) with C-terminal 18 amino acid deletion using Lipofectamine LTX Plus reagent (Invitrogen, USA) as per manufacturer’s protocol. A non-glycoprotein control (NE) was also generated using the same combination of plasmids as above, replacing plasmid encoding SARS-CoV2 spike, with 1 μg of an empty plasmid vector (pcDNA2.1).

Fourteen hours post-transfection, the medium was replaced with DMEM supplemented with 10% FCS and P/S and maintained at 37 °C with 5% CO_2_ for 3 days. The supernatant containing SARS-CoV-2 pseudo-virus particles and the non-glycoprotein control (NE) was spun down at 3000 × *g* for 10 min at 4 °C to remove cellular debris, aliquoted and stored at −80 °C. To validate the pseudo-virus activity, approximately 2 x 10^4^ cells/well of HEK293T were seeded on a black flat-bottomed 96-well plate (Corning, USA) precoated with poly-L-lysine and incubated overnight at 37 °C with 5% CO_2_. SARS-CoV-2 pseudo-virus particles and the non-glycoprotein control (NE) were diluted 1:5 and 1:20 in DMEM media supplemented with 10% FCS and P/S before infecting the cells with 100μL. HEK293T were incubated at 37 °C with 5% CO_2_ for 3 days. The intracellular luciferase level was measured on Varioskan LUX multimode microplate reader (ThermoFisher, USA) by replacing the medium with 50 μL Bright-Glo substrate (Promega, USA), as per manufacturer’s protocol. The results showed a high level of intracellular luciferase in HEK293T compared to the non-glycoprotein control (NE) validating this batch of pseudo-virus particle for SARS-CoV-2 used in this study (Supplementary Fig. 1). MDMi and Vero E6 were treated similarly with 1:5 dilution of Pseudo-virus particles stock and the level of intracellular luciferase was measured after 3 days.

### Plaque reduction neutralisation test (PRNT)

The neutralisation levels of a soluble recombinant angiotensin-converting enzyme 2 (ACE2) receptor against the SARS-CoV-2 (Wu) were verified by iPA [34]. The generation of a soluble recombinant version of human ACE2 protein was recently described [37].

### Purification and analysis of Nipah F-clamp and SARS-CoV-2 S-clamp proteins

SARS-CoV-2, termed S-clamp, Nipah F-clamp (GenBank: NP_112026.1) and the soluble hACE2 proteins were expressed in ExpiCHO cells and purified as previously described [37, 38]. Purified proteins were then characterised via SDS-PAGE, ELISA using ectodomain-specific monoclonal antibodies and size-exclusion chromatography. For SDS-PAGE, 4 µg of purified protein was mixed with DTT and analysed using a NuPAGE™ 4–12% Bis-Tris mini protein gel (ThermoFisher) as per the manufacturer’s instructions. Proteins were visualised by Coomassie staining.

For ELISA analysis, Nipah F-clamp, or SARS-CoV-2 S-clamp proteins were diluted to 2 µg/mL in PBS and coated overnight on Nunc MaxiSorp ELISA plates. The next day, plates were blocked with 150 µL/well of 5% KPL Milk Diluent/Blocking Solution Concentrate (SeraCare) in PBS with 0.05% Tween 20 (PBS.T) for 1 h at room temperature. Blocking buffer was removed, and serial dilutions of Nipah F- or SARS-CoV-2 S ectodomain-specific antibodies, 5B3 and CR3022 [39], were added. Plates were incubated for 1 h at 37 °C before they were washed three times with water and patted dry. Next, an HRP-conjugated goat anti-human secondary antibody (ThermoFisher) was added, and the plates incubated for 1 h at 37 °C. The plates were washed and dried as above before the addition of TMB Single Solution chromogen/substrate (Invitrogen). Plates were allowed to develop for 5 min at room temperature before the reaction was stopped by the addition of 2N H_2_SO_4_. Absorbance at 450 nm was read on a Varioskan LUX Multimode Microplate Reader (ThermoFisher). Data were analysed using GraphPad Prism version 8 using a one site – specific binding model.

Nipah F-clamp and SARS-CoV-2 S-clamp proteins were further analysed for their oligomeric state via size-exclusion chromatography using a Superdex 200 Increase 10/300 GL or Superose 6 Increase 10/300 GL column, respectively. Approximately 30–50 µg of protein in PBS was loaded onto the column using an ÄKTA pure FPLC system at a flowrate of 0.5 mL/min.

The limulus amebocyte lysate (LAL) based testing of endotoxin levels of the purified proteins were performed using Endosafe®-PTS^TM^ device and cartridges according to the manufacturer’s protocol (Charles River). Additionally, low endotoxin levels for the soluble hACE2, and monoclonal antibodies (3E8 and CO5) were also produced and validated by SDS-PAGE and ELISA. For ELISAs, 2 µg/mL in PBS of S-clamp or recombinant hACE2 or Hemagglutinin (HA) from influenza A H3 (Switzerland 2013) were coated overnight on Nunc MaxiSorp ELISA plates and then used to validate the hACE2, 3E8 and CO5 proteins respectively. The endotoxin levels were 1.1 EU/mg, 5.9 EU/mg, <5 EU/mg, <5 EU/mg and 124 EU/mg for SARS-CoV-2 S-clamp, Nipah F-clamp, hACE2, 3E8 and CO5 proteins, respectively.

### Preparation of fibrillar α-synuclein

Recombinant human α-synuclein monomer was obtained from Proteos, and in vitro fibril generation was performed with a final concentration of 2 mg/ml in phosphate-buffered saline (PBS) by incubation at 37 °C with agitation in an orbital mixer (400 rpm) for 7 days with daily cycles of sonication used to break down fibrillar aggregates as outlined previously [4]. The generation of fibrillar α-synuclein species was confirmed by transmission electron microscopy and Thioflavin T fluorescence prior to use.

### Western blotting

Primary microglial cells were collected and lysed using RIPA buffer (ThermoFisher). Proteins were separated in precast BioRad gradient (4–20%) gels. Proteins were then transferred to a nitrocellulose membrane and blocked for 1 h at room temperature (RT) using fluorescence western blocking buffer (Licor Bioscience). Membranes were washed 5 times with 5 min incubations per wash using either PBS containing 0.05% Tween-20 or tris(hydroxymethyl)aminomethane (Tris)-buffered saline containing 0.05% Tween-20. Primary human/rabbit/mouse antibodies, diluted in either Licor blocking buffer or 5% bovine serum albumin (BSA) solution, were then added to the membranes and incubated overnight at 4 °C. Glyceraldehyde 3-phosphate dehydrogenase (GAPDH) was used as loading controls. Following 5 washes, respective infrared-dye or horseradish peroxidase (HRP) conjugated secondary antibodies against primary antibodies was added to the membranes for 1 h at RT. Bands were visualised using either the Odyssey CLx imaging system (LI-COR) or enhanced chemiluminescence via SuperSignal^®^ West Pico Plus Chemiluminescent Substrate (Thermo-Scientific) accordingly to manufacturer’s instructions. Densitometric analysis was performed using Image Studio Lite software and the normalised band intensities were expressed as fold change over the control group.

### ELISA

Human IL-1 beta/IL-1F2 Duoset ELISA kit (R&D Systems, Catalog # DY201 was used to measure IL-1β in the supernatants of activated microglia. The assay was carried out according to the manufacturer’s instructions.

### MDMi immunocytochemistry

MDMi cells were fixed with 4% paraformaldehyde in PBS for 10 min. Cells were permeabilised with 0.1% triton X-100, subsequently washed three times in PBS, then blocked with 3% donkey serum in PBS for 1 h at room temperature. Following this, cells were incubated for 2 h at room temperature with combinations of the following antibodies: rabbit anti-TMEM (Cat# ab209064, Abcam), (1:100), rabbit anti-ASC (1:250), and mouse anti-Tubulin (Cat# T4026, Sigma), (1:250). Cells were then washed three times with PBS before incubating with secondary antibodies for 1 h at room temperature. Secondary antibodies used were donkey anti-rabbit 555 (1:2000) and donkey anti-mouse 488 (1:2000). Following three washes in PBS, cells were incubated with DAPI (1:4000 in PBS) before being mounted with Prolong gold antifade medium (Invitrogen) for fluorescent microscopy.

### Fluorescence microscopy methods

Images were collected using a Diskovery spinning disk confocal microscope (Andor/Nikon), 60XC CFI Plan Apochromat WI (NA 1.2) lens, with a disk pinhole size of 70um, and an Andor Zyla 4.2 sCMOS camera (Andor, UK). Images were collected at 12-bit and 2048 x 2048-pixel resolution. System settings, camera exposure times, and image brightness and contrast were consistent across all samples and optimised on Imaris 9.1.0 (Bitplane, UK) to create representative images for presentation. Samples were stained using the following fluorescent secondary antibodies: donkey anti-rabbit Alexa Fluor® 555, donkey anti-mouse Alexa Fluor® 488, and DAPI nuclear stain (brand). These fluorophores were then captured using laser lines 561 nm, 488 nm, and 405 nm for excitation with appropriate filters to maximise emission photon capture. Images were captured in a Z-stack for intracellular ASC speck visualisation, with a step size of 0.15 um.

### Statistical analysis

All data were analysed using Prism software (GraphPad 9.1.2). For growth kinetics, a multiple comparison using two-way ANOVA test with Sidak’s correction was used to compare within groups. Statistical significance was set at 95% (*p* = 0.05). For PRNT, a nonlinear regression with inhibitor vs. response (three parameters) model was used to determine the best-fit curve. Data are represented as mean +/− s.e.m. from at least 3 experiments, mice, or donors. ANOVA followed by Tukey’s post-test was performed to compare all treatment groups in densitometries and ELISA. *P < 0.05, **P < 0.01, and ***P < 0.001 denote statistically significant differences between indicated groups.

## Results

### Extensive microglial activation and NLRP3 inflammasome upregulation is observed in the brains of SARS-CoV-2 infected K18-ACE2 mice

**Fig. 1:**
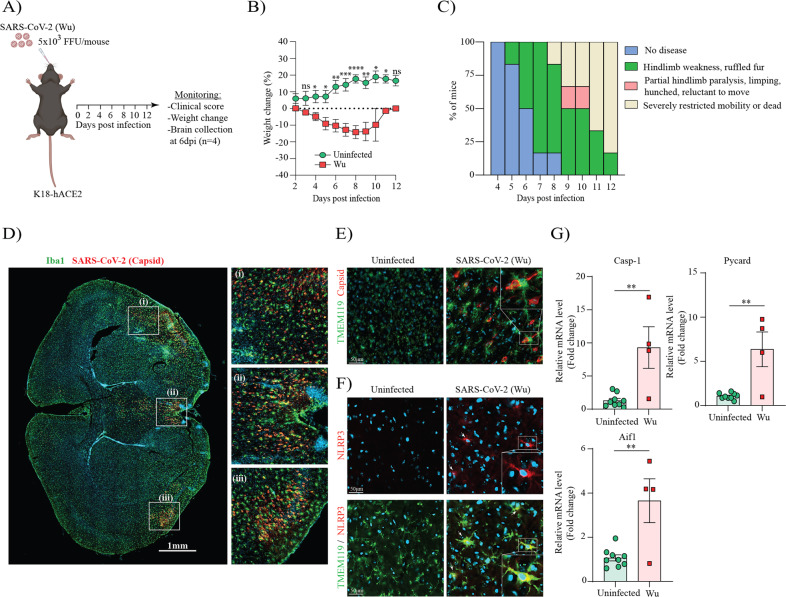
SARS-Cov-2 infected K18-hACE2 mice display virus spread in the brain with extensive microglial activation and NLRP3 inflammasome upregulation. Schematic representation for viral infection in (**A**). Percentage weight loss up to 12 days post infection (n=6 per group) in (**B**). Clinical score up to 12 days post SARS-CoV-2 infection (*n* = 6 per group) in (**C**). Representative SARS-CoV-2 infected brain at 6 dpi showing microglia marker Iba-1 (in green) and SARS-CoV-2 nucleocapsid (in red) and cell nuclei (in blue) assessed by immunofluorescence staining (*n* = 4 per group) in (**D**). Representative uninfected and SARS-CoV-2 infected brains showing microglial marker TMEM119 (in green) and SARS-CoV-2 nucleocapsid (in red) and cell nuclei (in blue) in (**E**). Representative uninfected and SARS-CoV-2 infected brains showing microglia marker TMEM119 (*n* = 4 per group) (in green) and NLRP3 (in red) and cell nuclei (in blue) in (**F**). Relative mRNA expression of Caspase-1 (Casp-1), Pycard (ASC) and Aif1 (Iba1) in uninfected and SARS-CoV-2 infected brains (*n* = 4–8 per group) in (**G**). Data points are means ± SEM from at least four mice per group. *P < 0.05, **P < 0.01, and ***P < 0.001 and ****P < 0.0001 by one-way analysis of variance (ANOVA) with Tukey’s post hoc test.

To study the impact of SARS-CoV-2 in the brain, female K18 hACE2 mice were infected by intranasal inoculation with an early clinical isolate “Wuhan” of SARS-CoV-2 (referred to herein as Wu). Clinical scores, body weights and survival were recorded up to 12 days post-infection (dpi) as illustrated in the scheme in Fig. 1A. SARS-CoV-2 infected mice began to lose weight from 4 dpi (Fig. 1B) and displayed hindlimb weakness and ruffled fur from 5 dpi (Fig. 1C). Notably, more than 75% of infected mice manifested severely restricted mobility or were deceased at 12 dpi (Fig. 1C), which is consistent with previous reports [40]. We next probed the brains of infected mice at 6 dpi by immunostaining for viral nucleocapsid protein using a nanobody we previously validated to detect divergent SARS-CoV-2 variants, including the ancestral Wu strain [29]. We identified extensive virus spread in the brain parenchyma of infected mice (Fig. 1D). Staining for the pan-microglia/macrophage protein, ionized calcium-binding adaptor molecule 1 (Iba-1) also revealed morphological alterations indicative of microglial activation in SARS-CoV-2 infected brains (Fig. 1D). To further confirm microglial involvement, we stained for the specific microglial marker TMEM119, which highlighted retracted thickened TMEM119-positive cell processes and large cell bodies in SARS-CoV-2 infected brains, characteristic of microglial activation (Fig. 1E). Interestingly, co-staining with anti-SARS-CoV-2 nucleocapsid, indicated that the virus is present in close proximity to these activated microglia (Fig. 1E). To determine whether inflammasome activation occurs upon SARS-CoV-2 infection in the brain, we assessed NLRP3 expression by immunofluorescence, showing a strong upregulation and colocalization in TMEM119-positive microglia, in comparison with uninfected mice (Fig. 1F). Inflammasome involvement in SARS-CoV-2 infected brains was also confirmed by qPCR, showing substantial upregulation for caspase-1, pycard (ASC) and Aif1 (Iba1) (Fig. 1G). Overall, these studies confirm that SARS-CoV-2 infection in mice leads to microglial activation and upregulation of NLRP3 inflammasome components.

### SARS-CoV-2 activates the inflammasome in human monocyte-derived microglia (MDMi) without supporting viral replication

**Fig. 2:**
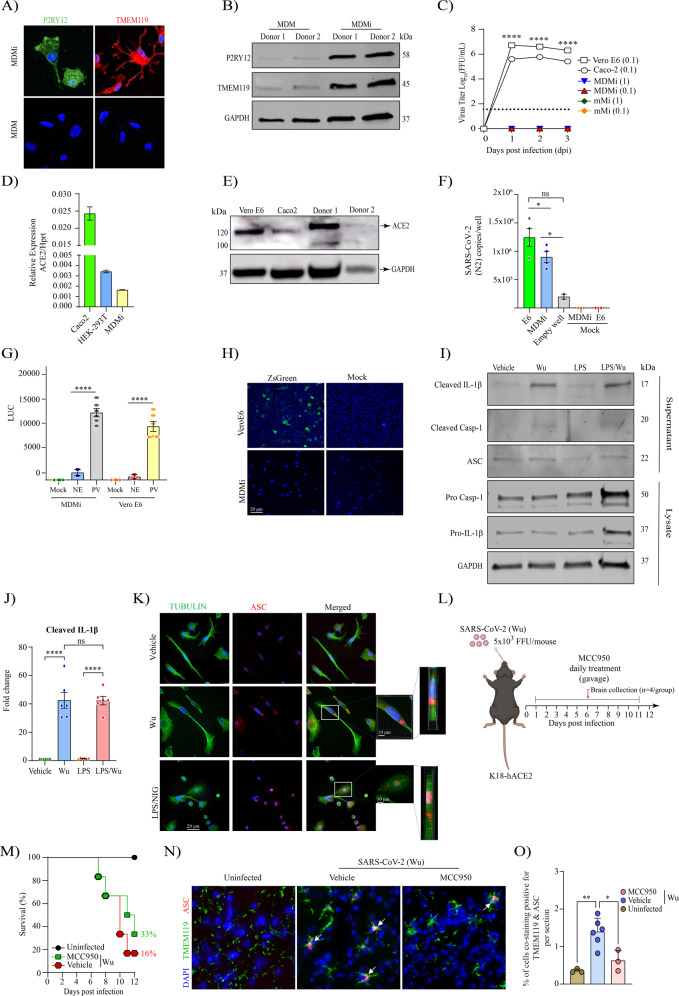
SARS-CoV-2 isolate activates the inflammasome in human monocyte-derived microglia (MDMi) in the absence of viral replication and can trigger microglial NLRP3 activation in vivo that is inhibited by MCC950. Microglia signature markers, P2RY12 and TMEM119 (in green) and cells nuclei (in blue) assessed by immunofluorescence staining, and representative western blot are presented in (**A**, **B**), respectively. Growth kinetics of SARS-CoV-2 (Wu) on MDMi, mouse microglia (mMi), Vero E6 and Caco2 cells in (**C**). Relative expression of ACE2 in MDMi by qPCR compared to Vero E6 and Hek-293T in (**D**). Level of ACE2 receptor in MDMi and mouse microglia compared to Caco2 and Vero E6 cells analysed by western blot shown in (**E**). Viral RNA levels from SARS-CoV-2 particles bound on cell surface expressed as N2 copies/well in (**F**) Intracellular luciferase level (LUC) delivered by pseudo-virus (PV) particle for SARS-CoV-2 in MDMi and Vero E6 compared to the non-glycoprotein control (NE) in (**G**). SARS-CoV-2 replication on MDMi (at MOI of 1) and Vero E6 (at MOI of 0.01) using SARS-CoV-2 reporter virus expressing ZsGreen fluorescent protein assessed directly under confocal microscopy at 3dpi are shown in (**H**). Western blot and densitometric analysis (fold change against vehicle group) for cleaved IL-1β, cleaved caspase-1 (p20), and ASC in the supernatants of LPS-primed or unprimed MDMi treated with SARS-CoV-2 (Wu) isolate for 24 h are presented in (**I**) and (**J**), respectively. Expression of GAPDH was determined in cell lysates. Immunofluorescence staining of MDMi treated with SARS-CoV-2 (Wu)–for Tubulin is stained in green and the formation of a characteristic inflammasome ASC speck (red) is shown in (**K**). LPS-Nigericin (Nig; 10 μM, 1 h) was used as a positive control. Scale bar, 20 μm. Inset magnified view of ASC specks. DAPI (blue), 4′,6-diamidino-2-phenylindole. Schematic representation for pharmacological intervention in (**L**). Percentage of survival up to 12 dpi on SARS-CoV-2, comparing uninfected, infected and MCC950-infected mice in (**M**). Representative uninfected, SARS-CoV-2 infected and MCC950-treated brains showing microglia TMEM119 (in green) and ASC (in red) and cells nuclei (in blue) assessed by immunofluorescence staining in (**N**). Percentage of cells from mouse brains co-staining positive for ASC and TMEM119 in (**O**). Data points are means ± SEM from at least three different donors or mice. *P < 0.05, **P < 0.01, and ***P < 0.001 and ****P < 0.0001 by one-way analysis of variance (ANOVA) with Tukey’s post hoc test (**C**, **J**) or two-way ANOVA test with Sidak’s correction (**D**, **F** and **G**).

Given our in vivo findings in mice, we next investigated the possible role of SARS-CoV-2 in promoting inflammasome activation in human microglia. We first generated MDMi following an established protocol to obtain adult microglia [41]. Initially, we verified that our generated MDMi highly expressed the typical microglia signature markers—P2RY12 and TMEM119 compared to monocyte derived macrophages (Fig. 2A, B). Next, we assessed whether these MDMi can support SARS-CoV-2 replication by monitoring infectious virus particle release after infection with multiplicity of infection (MOI) of 1 and 0.1. Notably, we found no secreted virus in the supernatant of infected MDMi and mouse primary microglia (mMi) cell culture supernatants, which contrasted to that in Vero E6 and Caco2 cells (Fig. 2C), supporting the notion that microglia cells do not support SARS-CoV-2 replication in vitro.

As angiotensin-converting enzyme 2 (ACE2) is the most well-characterised receptor for SARS-CoV-2 cell attachment [42], and is expressed in the CNS [43–46], we proceeded to determine the level of ACE2 in MDMi by qPCR and western blot. We observed that MDMi expressed ACE2 mRNA, although the levels were lower compared to Caco2 and HEK-293T cells (Fig. 2D). Western blot analysis from lysed microglia showed that ACE2 protein levels varied greatly in individual donors and displayed a differential pattern of expression compared to Vero E6 and Caco2 cells (Fig. 2E). The control cells (Vero E6 and Caco2) showed an expected full-length size of the glycosylated ACE2 form of approximately 120 KDa, while microglia cells showed molecular weights of ~135 and ~100 kDa. Notably, similar patterns have been also found in endothelial cells and heart tissue from COVID-19 patients [47, 48].

Given that MDMi expressed ACE2 receptor, yet did not support virus replication, we sought to determine whether SARS-CoV-2 binds to the microglial cell surface. To address this, MDMi were exposed to Wu at MOI of 1, incubated for 2 h at 4 °C and then subjected to several washes to remove all unbound particles. We found significant levels of viral RNA from bound virus particle on the cell surface (Fig. 2F), suggesting that SARS-CoV-2 can indeed bind to MDMi. We next investigated whether virus binding could promote viral entry by using a pseudo-virus entry assay as previously reported for SARS-CoV-2 [36, 49]. MDMi were transduced with SARS-CoV-2 pseudo-virus for 72 h and titre was determined by luciferase activity. We observed higher levels of intracellular luciferase activity in microglia cells infected with the pseudo-virus compared to the non-glycoprotein control (NE) and this level was comparable to pseudo-virus transduced Vero E6 cells (Fig. 2G), suggesting that SARS-CoV-2 has the ability to enter human microglia cells.

To further investigate whether SARS-CoV-2 productively replicates in MDMi, we utilised a recently characterised SARS-CoV-2 reporter virus bearing ZsGreen fluorescent protein [32], and infected cells using a MOI of 1 and monitored up to 3 days post infection (dpi). As expected, we detected a high level of ZsGreen fluorescent protein expression in Vero E6 cells (Fig. 2H and Supplementary Fig. 2). Furthermore, no intracellular ZsGreen fluorescence was observed in MDMi (Fig. 2H and Supplementary Fig. 2), confirming the inability of SARS-CoV-2 to establish replication in these cells.

Microglia are the resident immune cells found in the CNS that patrol the brain sensing for pathogens or damage-associated stress signal. To investigate microglial inflammasome activation in response to SARS-CoV-2, we exposed MDMi cells to SARS-CoV-2 and measured key inflammasome activation signals. MDMi were incubated with MOI of 1 of Wu isolate directly, or in LPS-primed cells. At 24-h post infection, western blot and immunocytochemistry was performed to examine markers of inflammasome activation (Fig. 2I–K).

We identified SARS-CoV-2 alone induced inflammasome activation in MDMi as measured by the release of cleaved IL-1β in the supernatant of cells exposed to Wu. This correlated with increased levels of cleaved caspase-1, validating activation of the inflammasome (Fig. 2I, J). These results were corroborated by ASC speck formation, a cellular hallmark of inflammasome activation. Increased ASC speck staining was observed in MDMi cells treated with Wu (both primed and unprimed), as well as LPS-primed cells activated with nigericin (Nig) as a positive control (Fig. 2K and Supplementary Fig. 3). Notably, our finding that SARS-CoV-2 exposure can directly activate the inflammasome in MDMi in the absence of priming, indicates that the virus can both prime and activate the inflammasome.

Having confirmed that microglial NLRP3 inflammasome is activated after SARS-CoV-2 infection in both human and mice models, we aimed to evaluate the therapeutic potential of blocking NLRP3 activation in vivo using the potent small-molecule NLRP3 inhibitor MCC950 [50]. To achieve this, we utilised the K18-hACE2 mouse COVID-19 model and started daily oral administration of MCC950 (20 mg/kg) 24 h after infection, and for up to 11 dpi as represented in the scheme in Fig. 2L. Notably, oral treatment with MCC950 increased survival in SARS-CoV-2 mice at 12 dpi (from 16% in untreated mice, to 33% in MCC950-treated mice) (Fig. 2M), although this was not statistically significant. We also obtained brains from a cohort of uninfected, and MCC950-treated and untreated SARS-CoV-2 infected mice at 6 dpi. Immunostaining these brains identified a significant decrease in the percentage of microglial ASC specks in MCC950-treated mice (Fig. 2O).

### SARS-CoV-2 spike protein activates the NLRP3 inflammasome in human microglia

**Fig. 3:**
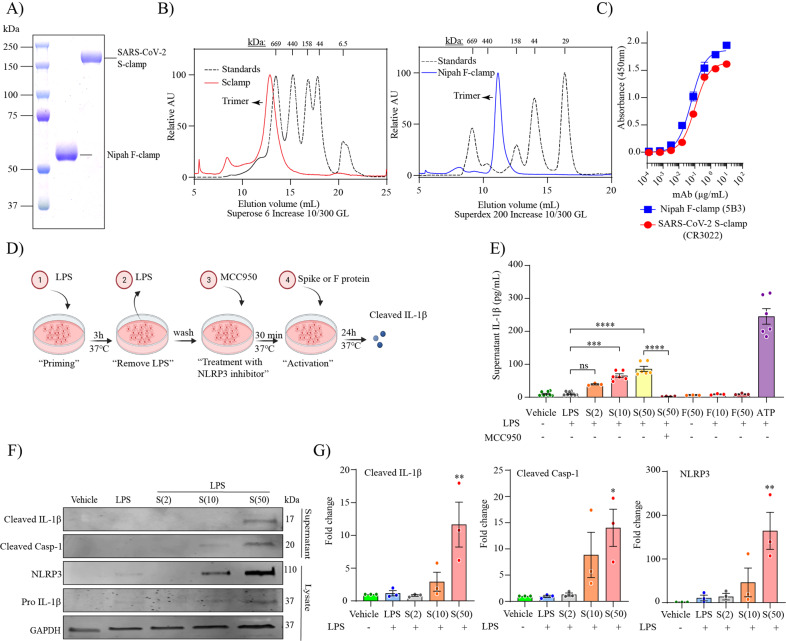
SARS-CoV-2 spike protein activates the NLRP3 inflammasome in LPS-primed MDMi. Prefusion-stabilized SARS-CoV-2 spike protein (S-clamp) and Fusion protein of Nipah virus (F-clamp) characterisation by SDS-PAGE (**A**), size-exclusion high-performance liquid chromatography (**B**) and ELISA with conformational specific monoclonal antibodies (**C**). Schematic representation for spike activation on LPS primed-MDMi (**D**). Spike–mediated microglial IL-1β secretion (supernatant) in vehicle (untreated) or LPS-primed MDMi exposed to S-clamp (S; 2–50 μg) or F-clamp (F; 50 μg) in presence or absence of MCC950 (10 μM) treatment is shown in (**E**). ATP (5 mM) treatment for 1 h was used as a positive control. Western blots (**F**) and densitometric analysis (fold change against vehicle group) (**G**) for NLRP3 in cell lysates of S-clamp–activated MDMi. Data are means ± SEM from at least three different donors. *P < 0.05, **P < 0.01, and ***P < 0.001 and ****P < 0.0001 by one-way analysis of variance (ANOVA) with Tukey’s post hoc test.

Given that MDMi cells express ACE2 and that live virus activated MDMi independent of viral replication, we next determined whether spike protein itself could trigger inflammasome activation directly in human microglia. We previously designed a prefusion-stabilized SARS-CoV-2 spike protein (S-clamp) that resembles a closed trimeric prefusion conformation [37]. To further identify the mechanism of inflammasome activation by SARS-CoV-2, we first produced low endotoxin S-clamp and a control trimeric fusion protein (F-clamp) from Nipah virus and validated these proteins using SDS-PAGE, size exclusion chromatography and ELISA (Fig. 3A–C). As expected, the monomeric molecular weight of the S-clamp and F-clamp monomers were 180 and 60 KDa, respectively (Fig. 3A). Additionally, we also confirmed that the majority of the S-clamp and F-clamp were presented in their trimeric form using size-exclusion chromatography and maintained reactivity assessed by binding of key specific antibodies (Fig. 3B, C). Next, we proceeded to expose LPS-primed MDMi with different concentrations of S-clamp or control F-clamp protein as shown in a schematic representation in Fig. 3D and identified that spike protein (S-clamp), but not the control F-clamp, induced significantly increased levels of IL-1β in supernatants after 24 h exposure (Fig. 3E). This activation was entirely ablated in the presence of MCC950, a selective inhibitor of NLRP3, confirming that the spike protein of SARS-CoV-2 is able to activate the NLRP3 inflammasome in LPS-primed microglia (Fig. 3E). We further confirmed spike-mediated inflammasome activation through western blotting, showing dose-dependent increases in cleaved IL-1β and cleaved caspase-1 in the supernatant, and NLRP3 in cell lysates (Fig. 3F, G). Although NLRP3 and pro-IL-1β are induced by LPS, they had evidently decayed after the wash-out of LPS but been re-induced by the spike protein. This suggests that spike protein is both priming and activating the inflammasome.

### Spike protein activates NLRP3 inflammasome through ACE2 in human microglia

**Fig. 4:**
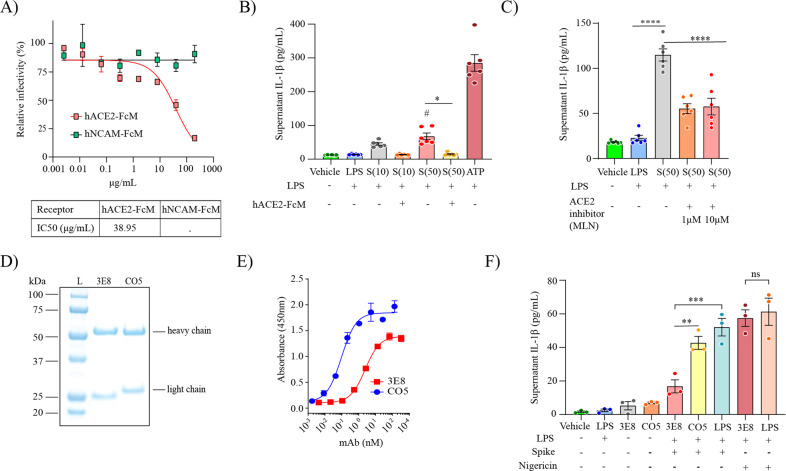
SARS-CoV-2 spike protein activates the NLRP3 inflammasome through ACE2 in MDMi. Relative of infectivity determined by Plaque Reduction Neutralisation Test (PRNT) to verify the neutralising level of a soluble receptor hACE2-FcM compared to a non-related SARS-CoV-2 receptor NCAM-FcM (top) and the inhibitory concentration (IC50) (bottom) (**A**). Spike–mediated IL-1β secretion (supernatant) in vehicle (untreated) or LPS-primed MDMIs exposed to S-clamp (S; 10–50 μg) in presence or absence of the soluble hACE2-FcM protein. ATP (5 mM) treatment for 1 h was used as a positive control (**B**). Inhibition of spike-mediated IL-1β secretion by ACE2 inhibition with MLN-4760 (1 or 10 μM) (**C**). Validation of low endotoxin anti-ACE2 (3E8) and anti-Hemagglutinin from influenza A H3 (CO5) proteins by SDS-PAGE and ELISA (**D**, **E**). Effect of 3E8 in blocking cells activation by spike protein in pre-treatment of LPS-primed MDMi exposed to S-clamp (**F**). Data are means ± SEM from at least three different donors. *P < 0.05, **P < 0.01, and ***P < 0.001 and ****P < 0.0001 by one-way analysis of variance (ANOVA) with Tukey’s post hoc test.

As spike protein is the major surface glycoprotein of the SARS-CoV-2 viral particle and contains a receptor- binding domain (RBD) that recognises ACE2 [51], we hypothesized that inflammasome activation is mediated by ACE2-RBD interaction. To address this, we first produced a low endotoxin version of a soluble human ACE2 protein (hACE2-FcM) and validated it in a neutralisation assay. We found that the soluble human ACE2 protein blocked SARS-CoV-2 entry into Vero E6 cells (Fig. 4A) with 50% inhibitory concentration (IC50) of 39 μg/mL, compared to control protein (NCAM-FcM) produced similar manner as hACE2-FcM (Fig. 4A). Based on this finding, we complexed the soluble ACE2 protein with the S-clamp at a molar ratio of 5:1, respectively, incubated at 37 °C for 1 h, and then used this complex to treat MDMi. We found a complete inhibition of IL-1β secretion in culture supernatants compared to S-clamp treatment alone (Fig. 4B). Additionally, pre-treatment of MDMi with the ACE2 inhibitor, MLN-4760, also significantly reduced spike protein induced IL-1β release from LPS-primed MDMi (Fig. 4C). Furthermore, to confirm that MDMi activation is ACE2 dependent, we used a well characterised monoclonal antibody (3E8), against human ACE2 [52]. To test the effect of 3E8 in blocking cell activation by spike protein, we first produced a low endotoxin level of 3E8 and a control antibody CO5 (anti HA of influenza A), followed by validation with SDS-PAGE and ELISA (Fig. 4D, E). Pre-treatment of LPS-primed MDMi with 3E8 specifically inhibited IL-1β secretion after activation with S-clamp compared to CO5 and nigericin (Fig. 4F), suggesting that spike-ACE2 interaction specifically contributes to inflammasome activation in microglia.

### SARS-CoV-2 spike protein primes the NLRP3 inflammasome through NF-κB

**Fig. 5:**
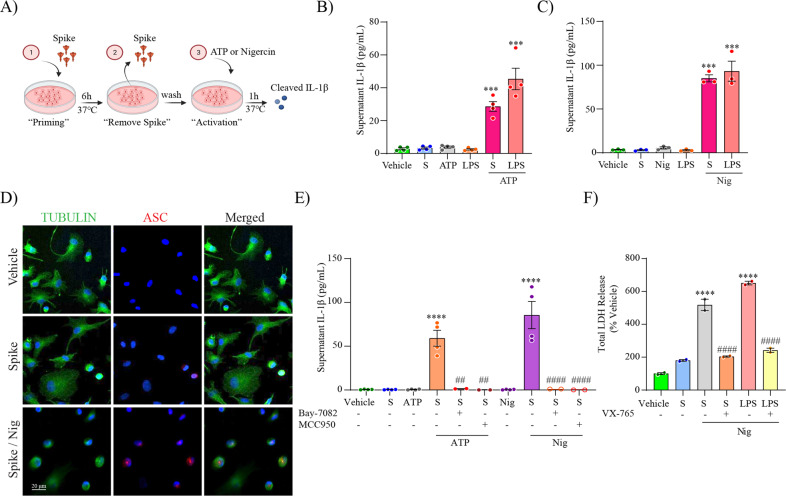
SARS-CoV-2 spike protein primes the NLRP3 inflammasome through NF-kB. Schematic representation for spike priming experiments (6 h) followed ATP or Nigericin (Nig) activation (1 h) (**A**). Level of secreted IL-1β in unprimed or S-clamp-primed MDMi (S; 50 μg 6 h) followed activation with ATP (5 mM, 1 h) in (**B**) or Nigericin (Nig; 10 μM, 1 h) in (**C**). In both LPS-primed cells were used as a positive control (200 ng/ml 3 h). Immunofluorescence staining of vehicle or S-clamp (S; 50 μg 6 h)–primed MDMi, activated with Nigericin (Nig; 10 μM, 1 h) showing tubulin (green) and the formation of a characteristic inflammasome ASC speck (red) are shown in (**D**). Scale bar 20 μm. ATP and Nigericin–mediated IL-1β secretion (supernatant) in vehicle (untreated) or S-clamp-primed MDMIs exposed to ATP (5 mM, 1 h) or Nigericin (Nig; 10 μM, 1 h) in presence or absence of Bay 11-7082 (3 μM) or MCC950 (10 μM) are shown in (**E**). Lactate dehydrogenase (LDH) release assay for quantification of caspase-1–dependent pyroptosis in S-clamp (S; 50 μg 6 h) primed cells activated with Nigericin (Nig; 10 μM, 1 h) in (**F**). LPS-Nigericin and VX-765 (20 μM) were used as positive controls. Data are means ± SEM from at least 3 independent donors. ***P < 0.001 and ****P < 0.0001 by one-way analysis of variance (ANOVA) with Tukey’s post hoc test.

We next evaluated if spike protein can also prime MDMi. To achieve this, MDMi were first stimulated with S-clamp for 6 h, then media containing S-clamp was removed and replaced with fresh media and incubated with either ATP or nigericin for 1 h as the inflammasome-activating signal, as shown in a schematic representation in Fig. 5A. We observed a significant IL-1β release for both activators in S-clamp-primed cells, in comparison with either vehicle, S-clamp, ATP or nigericin alone, and the level was of a similar, but slightly reduced magnitude to LPS-primed cells (Fig. 5B, C). We also confirmed an S-clamp priming effect through immunocytochemistry, showing ASC speck formation in S-clamp-primed cells activated with nigericin (Fig. 5D), displaying a similar morphology to cells previously primed with LPS and activated with nigericin used as a positive control (Fig. 2K).

To test if NF-kB signalling pathway is required for inflammasome priming by spike protein, we pre-treated MDMi with the NF-kB inhibitor, Bay 11-7082, before stimulation with S-clamp and the addition of ATP or nigericin. We found a complete inhibition of IL-1β release (Fig. 5E), confirming S-clamp priming activity is mediated through the NF-kB pathway. Moreover, after priming with S-clamp, and before activating with either ATP or nigericin, we treated the cells with the NLRP3 inhibitor MCC950 and demonstrated a complete inhibition of IL-1β secretion (Fig. 5E), confirming as expected that nigericin and ATP triggered NLRP3 after priming by spike protein.

Recently, it has been shown that SARS-CoV-2 triggers pyroptosis in human monocytes [53]. Therefore, we assayed whether S-clamp priming and nigericin inflammasome activation triggered pyroptosis in MDMi. Pyroptosis was quantified using lactate dehydrogenase (LDH) release, and the caspase-1 inhibitor VX-765 was used to selectively assess the role for inflammasome activation in cell death. We observed that nigericin treatment of S-clamp primed MDMi readily triggered caspase-1-dependent pyroptosis within 1 h (Fig. 5F) which was significantly reduced in the presence of caspase-1 inhibitor VX-765. SARS-CoV-2 spike protein mediating priming of the inflammasome has recently been documented in macrophages derived from COVID-19 patients [54]. Our data now provides strong evidence that SARS-CoV-2 spike protein can also prime the NLRP3 inflammasome through the NF-kB pathway in human microglia.

### SARS-CoV-2 promotes α-synuclein mediated NLRP3 inflammasome activation by priming MDMi through spike protein

**Fig. 6:**
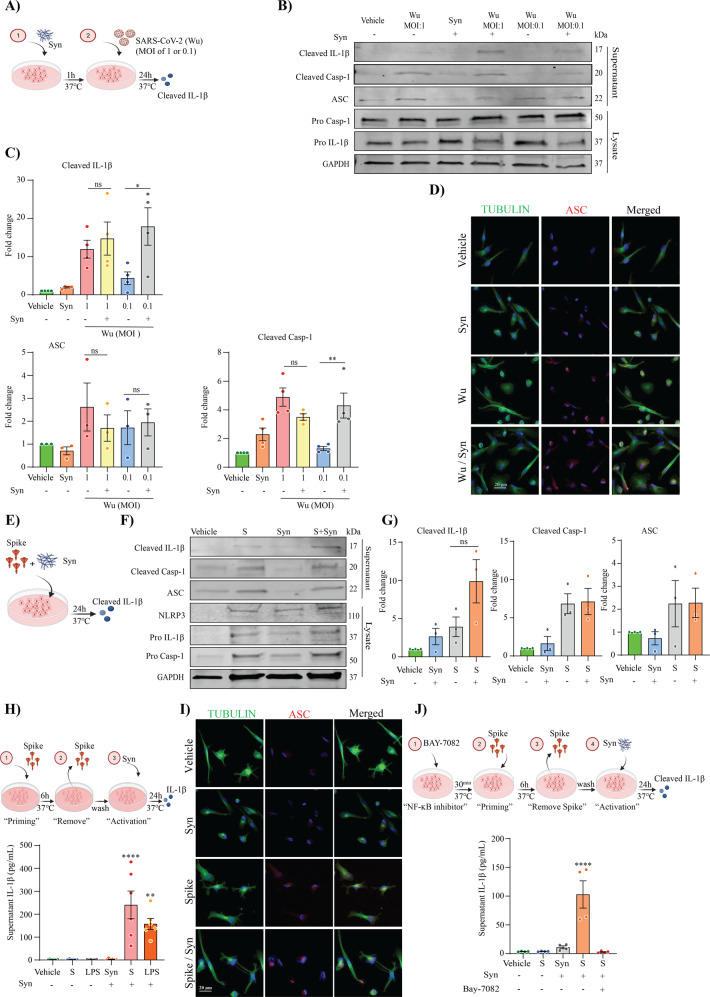
SARS-CoV-2 promotes α-synuclein mediated NLRP3 inflammasome activation, priming MDMi through spike protein. A schematic representation of SARS-CoV-2 exposure in presence of α-synuclein (Syn) for 24 h on MDMi (**A**). Level of cleaved IL-1β, in the supernatants of MDMi, treated with either Wu (MOI 1, 0.1) or α-synuclein (Syn; 10 μM) or together for 24 h by western blot and densitometric analysis are shown in panel (**B**, **C**), respectively. Expression of GAPDH was determined in cell lysates. Immunofluorescence staining of MDMi treated with either α-synuclein (Syn; 10 μM) or Wu (MOI 0.1) or together for 24 h showing tubulin (green) and the formation of a characteristic inflammasome ASC speck (red) are shown in (**D**). Scale bar, 20 μm. A schematic representation of spike (S-clamp) exposure in presence of α-synuclein (Syn) for 24 h on MDMi (**E**). Western blots and densitometric analysis (fold change against vehicle group) for cleaved caspase-1 (p20), cleaved IL-1β, and ASC in the supernatants of MDMi treated with either s-clamp (S; 50 μg) or α-synuclein (Syn; 10 μM) or together for 24 h are presented in **F** and **G**. A Schematic representation for spike priming (6 h) followed α-synuclein (Syn) activation for 24 h in **H** (top) and α-synuclein (Syn; 10 μM) –mediated IL-1β secretion (supernatant) in unprimed or S-clamp-primed MDMi (S; 50 μg 6 h) is shown in ** H** (bottom). LPS-primed cells were used as a positive control (200 ng/ml 3 h). Representative immunofluorescence of S-clamp (spike; 50 μg 6 h) primed MDMI activated with α-synuclein (Syn; 10 μM) for 24 h showing staining for Tubulin (green) and ASC speck formation (red) in **I**. A schematic representation for NF-kB inhibition on spike-primed MDMi (6 h) followed α-synuclein (Syn) activation for 24 h (**J**, top). IL-1β secretion in vehicle (untreated) or S-clamp-primed MDMIs exposed to α-synuclein (Syn; 10 μM) in presence or absence of Bay11-7082 (3 μM) for 24 h in **J** (bottom). Data are means ± SEM from at least three different donors. *P < 0.05, **P < 0.01 and ****P < 0.0001 by one-way analysis of variance (ANOVA) with Tukey’s post hoc test.

The underlying mechanism of microglial activation by SARS-CoV-2 and their impact in presence of endogenous neurodegenerative disease-driving triggers are unclear. To better understand the effect of SARS-CoV-2 infection on promoting human microglia activation in relation to brain disease triggers, MDMi were incubated with Wu (MOI 0.1 or 1) in the presence or absence of preformed fibrils of α-synuclein for 24 h as shown in a schematic representation in Fig. 6A, and the level of cleaved IL-1β, cleaved caspase-1 and ASC in the supernatant were measured by western blot. We identified the presence of cleaved IL-1β, cleaved caspase-1, and ASC in the supernatant of MDMi treated with SARS-CoV-2 and α-synuclein, in the absence of LPS (Fig. 6B). Notably, an increase of inflammasome activation was achieved with Wu (0.1 MOI) in presence of α-synuclein, whereas neither Wu at MOI 0.1, or α-synuclein alone, were able to activate the inflammasome; however, when combined, they released significant cleaved IL-1β, and cleaved caspase-1 in the supernatant (Fig. 6B, C). Immunocytochemistry for ASC further confirmed this observation, showing increased levels ASC speck formation in cells treated with Wu at MOI 0.1 in presence of α-synuclein (Fig. 6D).

To examine the role of SARS-CoV2 spike protein in the context of α-synuclein microglial inflammasome activation, we repeated the experimental paradigm with spike in presence of α-synuclein for 24 h as shown in a schematic representation in Fig. 6E. Performing western blot for cleaved IL-1β, cleaved caspase-1 and ASC on supernatants, we confirmed inflammasome activation with both spike and α-synuclein in MDMi, and a trend towards an increase in cleaved IL-1β when α-synuclein and spike were combined (Fig. 6F, G). We next proceeded to evaluate whether spike protein could prime MDMi for enhanced inflammasome activation driven by α-synuclein. We therefore primed MDMi with S-clamp for 6 h followed by α-synuclein activation for 24 h (Fig. 6H). We confirmed by ELISA that no significant IL-1β release was induced by either S-clamp priming or α-synuclein when administered alone, however robust IL-1β release was found in S-clamp primed cells in the presence of α-synuclein, at levels even greater than LPS-primed cells (Fig. 6H). This result correlated with ASC speck formation in S-clamp primed cells activated with α-synuclein (Fig. 6I). To confirm the S-clamp priming effect in this context was also mediated through NF-kB signalling, we pre-treated cells with the NF-kB inhibitor Bay 11-7082 and demonstrated a complete inhibition of cleaved IL-1β release (Fig. 6J). Altogether, our data demonstrates that SARS-CoV-2, and spike protein, can both prime and activate the NLRP3 inflammasome in human microglia, also potentiating activation in the presence of α-synuclein, supporting a possible risk factor for COVID-19 in Parkinson’s disease and neurodegeneration.

## Discussion

Several recent clinical studies have documented increased inflammasome activity in response to SARS-CoV-2 infection, leading to immune dysregulation that is associated with COVID-19 severity [55, 56]. In the periphery, it has been observed that monocytes from COVID-19 patients have increased inflammasome activation and undergo pyroptosis, which is associated with higher levels of plasma IL-1β in critically ill patients [53, 55], and the presence of ASC speck formation in lung macrophages [56].

In the CNS, a plurality of evidence has shown microglial activation in COVID-19 deceased patients, with enlargement of cell soma, and thickening of processes detected by staining for microglial markers [12, 13, 57]. The colocalization of SARS-CoV-2, ACE2, and NLRP3 within neurons, astrocytes, and microglia was also found in cerebral cortical tissue in three post-mortem COVID-19 cases [58]. Similarly, microglial activation has been confirmed in multiple preclinical in vivo models of COVID-19 [14–16]. Aligned with these results, here we show for the first time that SARS-CoV-2 invasion in the brain following intranasal infection of K18-hACE2 mice, leads to extensive microglial NLRP3 inflammasome activation.

While the ability of SARS-CoV-2 to enter the nervous system and to infect and replicate in CNS cells has been studied extensively, there are contradicting findings [59]. A remaining debate is whether the observed neurological manifestations in COVID-19 are attributed to virus invasion into the brain, or as a consequence driven by systemic factors or comorbidity. There is currently no conclusive evidence on whether SARS-CoV-2 can readily cross the blood-brain barrier (BBB) in humans, however SARS-CoV-2 antigens (such as spike protein) have been found in cortical neurons from patients who died of COVID-19 [60]. Interestingly, our findings also show that spike protein alone can prime and activate microglial inflammasomes (Fig. 3) supporting a mechanistic role for viral protein ‘spill-over’ into the CNS. In addition, SARS-CoV-2 genetic material and viral replication was recently reported in astrocytes in the brains of five individuals who died from COVID-19 presenting with brain damage [61]. Moreover, in vivo experiments performed in hamsters provide evidence that the basement membrane of the BBB is disrupted after SARS-CoV-2 infection, supporting that SARS-CoV-2 can cross the BBB in a transcellular pathway [62]. The capacity of SARS-CoV-2 to disrupt the blood-CSF barrier has also been demonstrated using human brain organoids [63], and human induced pluripotent stem cell-derived brain capillary endothelial-like cells [64], supporting that SARS-CoV-2 can use this route to enter the CNS.

Apart from the BBB, there is evidence demonstrating that SARS-CoV-2 can enter the CNS through cranial nerves that innervate the olfactory mucosa [65], as well through the vagus nerve to the brainstem, by immunohistochemical detection of SARS-CoV-2 in vagus nerve fibres [66]. Collectively, although it is likely that the ability of SARS-CoV-2 to readily invade the CNS is limited, there is enough evidence to suggest that this can happen at least in a subset of the COVID-19 infected population. Building on these observational studies, here we provide mechanistic insight into the molecular requirements of SARS-CoV-2 inflammasome activation in human microglial cells.

SARS-CoV-2 entry into host cells has been thoroughly described and is mediated by the binding of viral spike protein to the human receptor ACE2 [67]. The expression of ACE2 receptor in the normal brain shows contradictory findings, with studies demonstrating no or low protein expression in the brain using tissue microarrays in a body-wide analysis [68]. However, more recently, using a collection of available datasets at the mRNA level, relatively high levels of ACE2 were found in the choroid plexus and paraventricular nuclei of the thalamus [43], which was confirmed at the protein level, through immunohistochemical staining for ACE2 in the choroid plexus and ependymal cells [69]. Interestingly, in COVID-19-affected brains, ACE2 expression is upregulated in endothelial cells in the white matter, with a correlation of higher expression in patients with more severe neurological symptoms [69].

The expression of ACE2 in the diseased brain has also been studied in the context of neurodegenerative diseases, with upregulated ACE2 expression observed in Alzheimer’s disease brains [46]. In the context of PD, angiotensin type-1 receptor (AT1) and ACE2 autoantibodies were found elevated in PD patients compared with age-matched controls in serum and in cerebrospinal fluid (CSF), suggesting that dysregulation of renin-angiotensin autoantibodies could contribute to PD progression [70].

Notably, in relation to the present study, the development of autoantibodies against ACE2 [71] and AT1 [72] has been also found after SARS-CoV-2 infection. Our results show that MDMi microglial cells express ACE2 receptor and, although the level is relatively low, SARS-CoV-2 is able to enter these cells but does not establish viral replication. This has also been shown for human in vitro differentiated myeloid dendritic cells (mDC) as well as M1 and M2 macrophages, where in contrast to Vero E6 controls, no infectious virus production of SARS-CoV-2 is observed up to 48 h after inoculation [73]. One limitation of the MDMi cells used in our study, is the potential for residual characteristics to be carried over from their monocyte precursors [41]. Notably however, our data in MDMi cells aligns with the work of Yang et. al. where it was demonstrated that human pluripotent stem cell (hPSC)-derived microglia also express ACE2 receptor and are permissive to SARS-CoV-2-pseudo-virus entry [49]. Additionally, this study also found low or undetectable levels of viral RNA in hPSC-derived microglia exposed to infectious SARS-CoV-2, offering further evidence that while ACE2 mediated viral uptake is possible, hPSC-derived microglia do not support SARS-CoV-2 replication [49].

We also show that SARS-CoV-2 can activate the inflammasome in human microglia, through the read-out of cleaved IL-1β, cleaved caspase-1, and ASC speck formation in the supernatant. As it has been previously demonstrated that the interaction between ACE2 receptor and spike protein can induce the hyperactivation of NLRP3 in endothelial cells [74], we investigated the role of spike-ACE2 interaction relative to NLRP3 activation in microglia using a prefusion-stabilized SARS-CoV-2 spike protein (S-clamp) [37]. To confirm that NLRP3 activation on MDMi was ACE2 dependent we used a soluble human ACE2 receptor (hACE2-FcM), an ACE2 inhibitor (MLN-4760), and a well characterised monoclonal antibody (3E8) [52]. All three approaches confirmed that spike protein can activate NLRP3 in human microglia-like cells through ACE2.

Although we demonstrated that spike protein can activate the NLRP3 inflammasome in human microglia, it is worth noting that SARS-CoV-2 also encodes other viral proteins that could be involved in inflammasome activation. SARS-CoV-2 is comprised of a nucleocapsid protein (N), spike protein (S), membrane protein (M), and envelope protein (E), in addition to a series of accessory proteins (ORF3a, ORF6, ORF7a, ORF7b, ORF8, and ORF10). Previous studies with the original SARS coronavirus, SARS-CoV have shown that protein E and ORF3a activate NLRP3, forming multimeric complexes that act as ion channels activating the NLRP3 inflammasome with IL-1β release, driven through NF-kB [75–77]. Moreover, recent evidence demonstrated that N-protein interacts directly with NLRP3, promoting the binding of NLRP3 with ASC, facilitating NLRP3 inflammasome assembly indicating another distinct mechanism of direct inflammasome activation through interaction of a viral protein with NLRP3 [78]. Our findings now provide further information that SARS-CoV-2 spike protein contributes directly to activating the NLRP3 inflammasome through ACE2.

Priming of the inflammasome in cells is a process necessary to induce transcriptional up-regulation of NLRP3 and pro-IL-1β [79]. Our initial observation that the SARS-CoV-2 virus itself can trigger inflammasome activation in MDMi without the need for priming supports a role for vigorous virus-mediated inflammasome activation in vivo. We also confirmed that spike protein alone can prime the inflammasome through NF-kB in MDMi, allowing for NLRP3 activation with classical inflammasome activators ATP and nigericin, as has been previously reported in human monocytes, macrophages, and human lung epithelial cells [80, 81]. These findings support that human coronavirus spike protein can induce innate immune responses through NF-kB signalling.

We previously documented that activation of microglial NLRP3 inflammasomes through α-synuclein fibrils is a major driver of dopaminergic neuronal loss in experimental PD [4]. The accumulation of α-synuclein aggregates, as seen in Lewy bodies, and their spread throughout the brain is correlated with the stages of PD progression [82]. Of importance to the present study, there are increasing reports of significant neurological complications from SARS-CoV-2 infection in human patients [12, 13, 17, 23, 60, 61, 83, 84]. The correlation between viral infection and the manifestation of Parkinson-like symptoms has been described for a variety of viruses including influenza virus, JEV, and West Nile virus (WNV) infection resulting in tremor, myoclonus, rigidity, bradykinesia, and postural instability [85]. The activation of microglial NLRP3 inflammasome in the brain has also been demonstrated after viral infection with JEV [7] and WNV [86]. Indeed, we utilised WNV-infected C57BL6/J mice in this study as a control for our SARS-CoV-2 infected K18-hACE2 mice, which similarly demonstrated upregulated microglial NLRP3 inflammasome activation (Supplementary Fig. 4).

Moreover, post-mortem analysis performed on WNV-infected individuals showed an increased level of α-synuclein [87]. This finding prompted the hypothesis that α-synuclein is upregulated during infection as an antiviral factor in neurons, where it is proposed to act as a natural antimicrobial peptide to restrict viral infection in the brain [87, 88]. However, a recent study indicated that there were no alterations in α-synuclein levels in serum and CSF of COVID-19 patients with neurological symptoms [89]. These findings suggest that the reported cases of parkinsonism after SARS-CoV-2 infection could be a consequence of an increased proinflammatory environment, mediated by blood brain barrier (BBB) disruption [90], peripheral cell infiltration [91], and microglial activation [92]. These processes could be enhanced in the presence of ongoing synucleinopathies, or risk factors such as aging and poor health.

In addition, a recent study demonstrated that *NLRP3* inflammasome genetic variants are associated with critical disease in severe COVID-19 patients, especially in elderly male individuals with reduced sickness symptom complex (SSC) and with increased body mass index (BMI), hypertension, and diabetes type 2 [93]. In conjunction, all these factors could lead to an accelerated neuronal loss, correlated with the reported parkinsonism symptoms and possible susceptibilities to developing PD post-SARS-CoV-2 infection.

Here we addressed the impact of SARS-CoV-2 on microglia in presence of α-synuclein. We showed that SARS-CoV-2 promotes α-synuclein mediated NLRP3 inflammasome activation by priming MDMi through spike protein, providing ex vivo support for the negative impact of SARS-CoV-2 on neurodegenerative diseases such as PD. It is also worth noting that there are several lines of evidence in the literature indicating neurological complications resulting from SARS-CoV-2 infection. These include: (i) Possible cases of neuroinvasion by SARS-CoV-2 as observed in humans and preclinical COVID-19 models [12, 56, 60, 94, 95]; (ii) Extended microglial activation with pronounced neuroinflammation as reported in brain autopsies obtained from deceased SARS-CoV-2 patients [12, 13, 96]; (iii) Significant deterioration of motor performance and motor-related disability in PD patients recovering from COVID-19 [97, 98]; and (iv) Accumulation of hyperphosphorylated Tau and α-synuclein occurring beyond viral clearance in SARS-CoV-2 infected hamsters [15] and Lewy body formation in SARS-CoV-2 infected macaques [99].

Thus, our finding complements the knowledge-gap in molecular mechanisms by which SARS-CoV-2 may activate microglia and lead to neurological manifestations. Our data suggest that the spike protein-mediated priming and/or activation of microglia through the ACE2-NF-kB axis may promote NLRP3 inflammasome activation leading to neuroinflammation and neurological phenotypes. Further, this process may be enhanced in the presence of neurodegenerative disease triggers such as α-synuclein aggregates, supporting a possible role for COVID-19 in triggering brain diseases such as PD. Pharmacological inhibition of the NLRP3 inflammasome upon SARS-CoV-2 infection can suppress immune overactivation and alleviate COVID-19 in preclinical mice models [100]. Here, we further highlight the therapeutic potential of inhibiting NLRP3-driven microglial activation in the COVID-19 brain using an oral drug approach with the brain-penetrant small molecule MCC950 [4]. Since NLRP3 inhibitors are currently in clinical development for neurodegenerative diseases, including PD [4, 101], these findings also support a potential therapeutic avenue for treatment of SARS-CoV-2 driven neurological manifestations.

### Supplementary information

Supplementary material
