# Supplementary material for: SARS-CoV-2 drives NLRP3 inflammasome activation in human microglia through spike protein
Source: Mol Psychiatry. 2022 Nov 1;28(7):2878–93. doi: 10.1038/s41380-022-01831-0 (PMC10615762; doi:10.1038/s41380-022-01831-0)
Supplement: Supplementary file 1 — Supplementary material [file 41380_2022_1831_MOESM1_ESM.docx]

**SUPPLEMENTARY FIGURES**

**
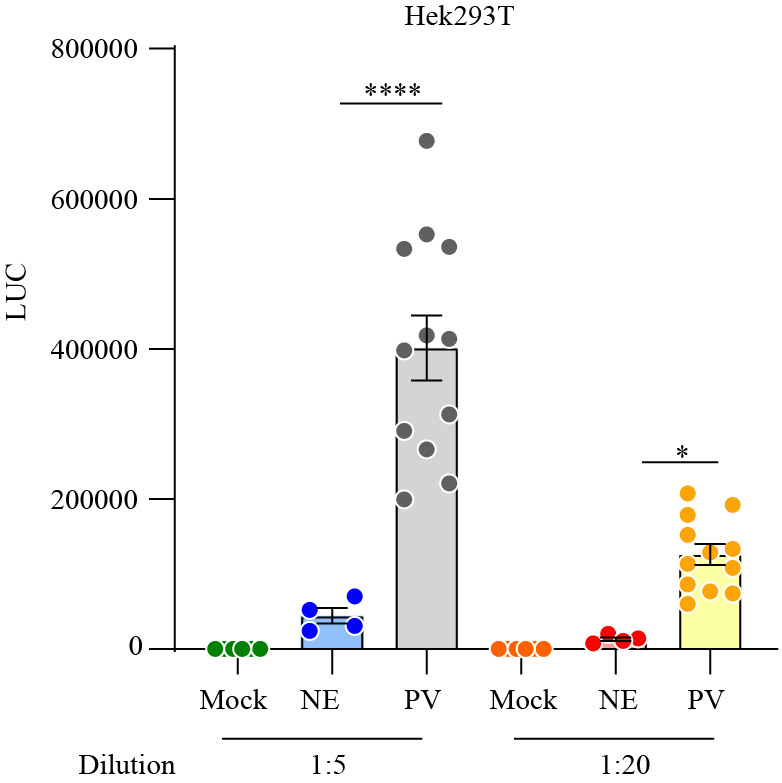
**

**Supplementary Figure 1.** Validation of Intracellular luciferase level (LUC) delivered by pseudo-virus (PV) particle for SARS-CoV-2 in HEK293T compared to the non-glycoprotein control (NE). Data are means + SD from one experiment. *P < 0.05, **P < 0.01, and ***P < 0.001 and **** P < 0.0001 by one-way analysis of variance (ANOVA) with Tukey’s post hoc test.

**
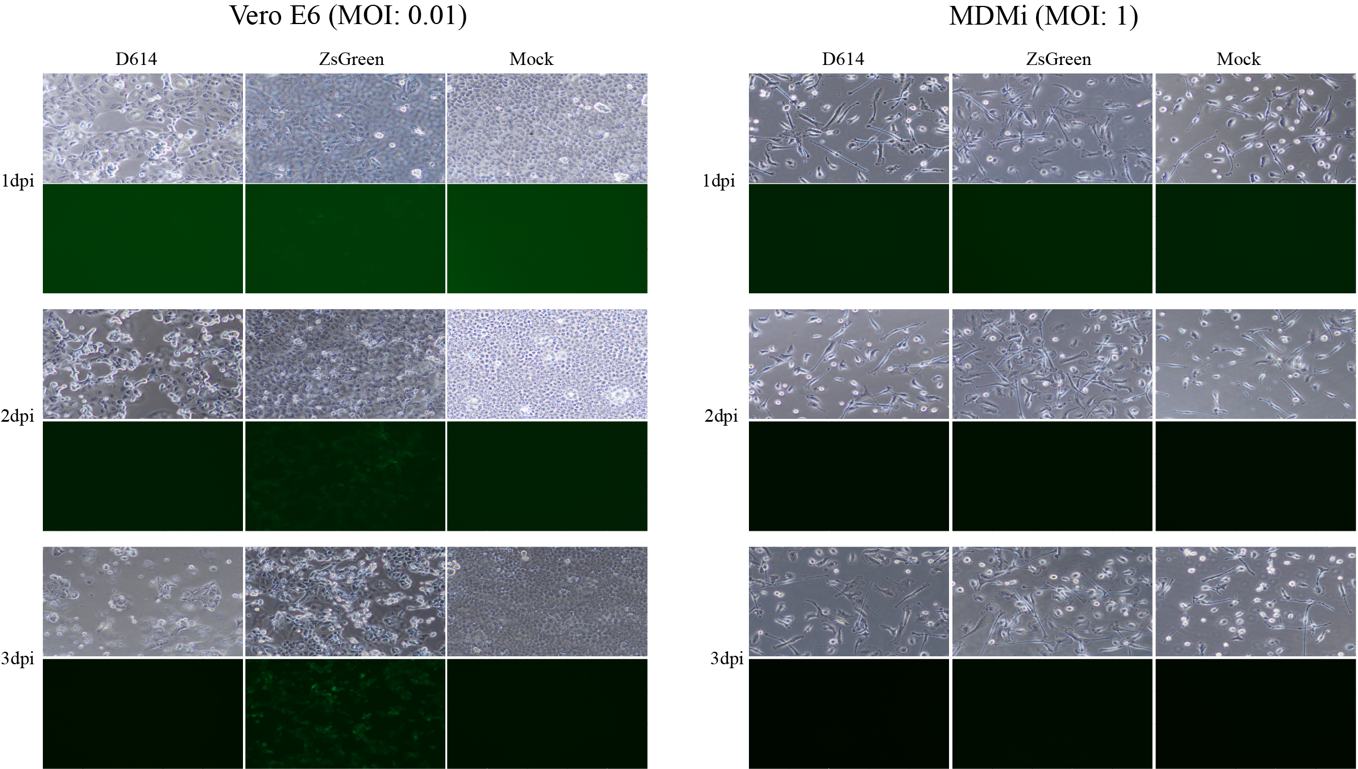
**

**Supplementary Figure 2.** SARS-CoV-2 replication on MDMi (at MOI of 1) and Vero E6 (at MOI of 0.01) using SARS-CoV-2 reporter virus expressing ZsGreen fluorescent protein compared to WT virus (D614) assessed directly under microscopy. Images on the left and right panel are from infected Vero E6 and MDMi cell, respectively. Images for each time point were taken under bright-field (on top) and with the fluorescence filter (on bottom) using 40x magnification.


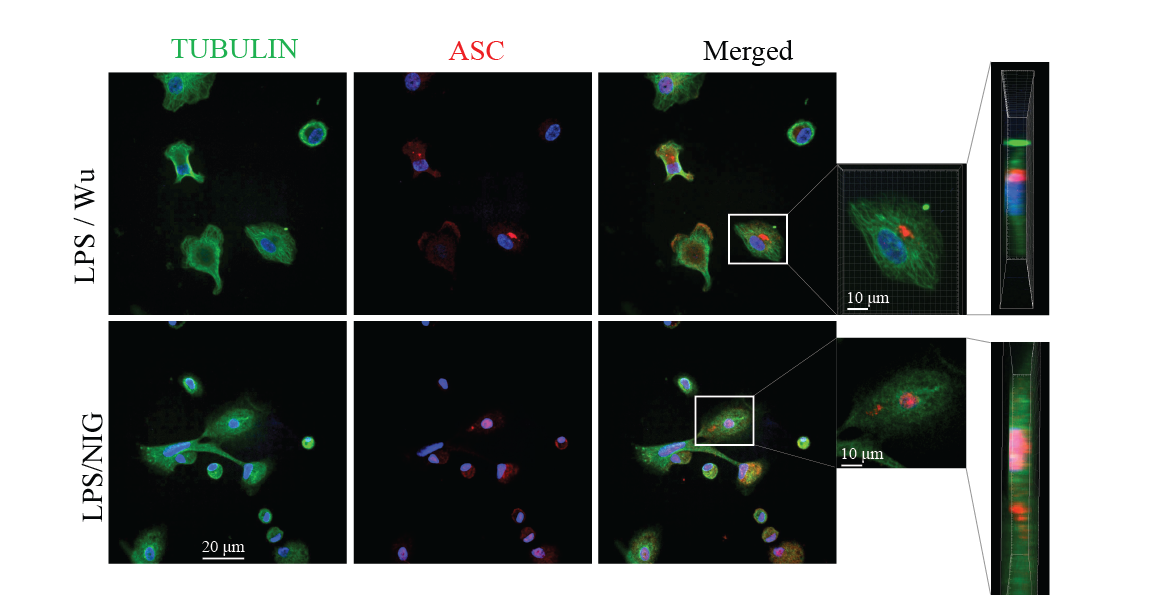


**Supplementary Figure 3.** SARS-CoV-2 activates NLRP3 inflammasome in LPS-primed MDMi. Immunofluorescence staining of LPS-primed MDMi treated with SARS-CoV-2 (Wu)–for Tubulin is stained in green and the formation of a characteristic inflammasome ASC speck is shown in red. LPS-Nigericin (Nig; 10μM, 1 hour) was used as a positive control. Scale bar, 20 μm. Inset magnified view of ASC specks. DAPI (blue), 4′,6-diamidino-2-phenylindole.

**
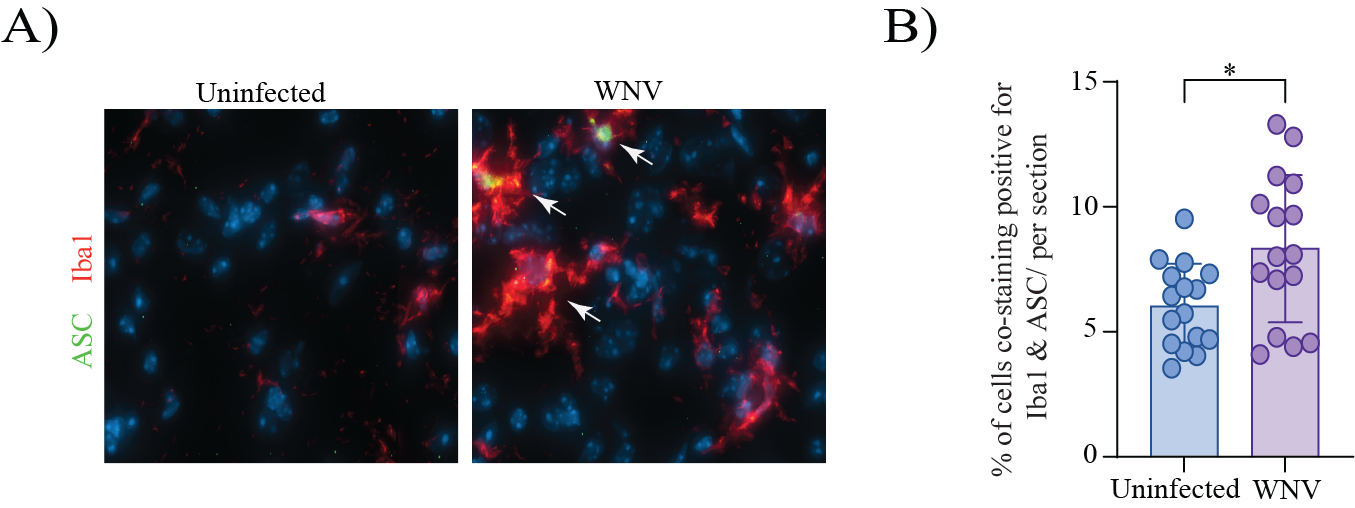
**

**Supplementary Figure 4.** West Nile Virus (WNV) infection triggers microglial- NLRP3 activation in mice. Representative uninfected and WNV infected brains showing microglia marker Iba1 (n=3 per group) (in red) and ASC (in green) and cell nuclei (in blue) in (A). Percentage of cells from mouse brains co-staining positive for Iba1 and ASC in (B). Data points are means + SEM *P < 0.05.
